# Hippocampal volumes in patients exposed to low-dose radiation to the basal brain. A case–control study in long-term survivors from cancer in the head and neck region

**DOI:** 10.1186/1748-717X-7-202

**Published:** 2012-11-29

**Authors:** Erik Olsson, Carl Eckerström, Gertrud Berg, Magnus Borga, Sven Ekholm, Gudmundur Johannsson, Susanne Ribbelin, Göran Starck, Anna Wysocka, Elisabet Löfdahl, Helge Malmgren

**Affiliations:** 1 Institute of Neuroscience and Physiology;, Sahlgrenska University Hospital, Sahlgrenska Academy, University of Gothenburg, Gothenburg, Sweden; 2 Department of Oncology, Sahlgrenska University Hospital, Sahlgrenska Academy, University of Gothenburg, Gothenburg, Sweden; 3 Department of Biomedical Engineering, Linköping University, Linköping, Sweden; 4 Department of Imaging Sciences, University of Rochester Medical Center, Rochester, N.Y, USA; 5 Department of Endocrinology, Sahlgrenska University Hospital, Sahlgrenska Academy, University of Gothenburg, Gothenburg, Sweden; 6 Department of Radiation Physics, Sahlgrenska University Hospital, Sahlgrenska Academy, University of Gothenburg, Gothenburg, Sweden; 7 Department of Medical Physics and Biomedical Engineering, Sahlgrenska University Hospital, Sahlgrenska Academy, University of Gothenburg, Gothenburg, Sweden; 8 Department of Philosophy, Linguistics and Theory of Science, University of Gothenburg, Gothenburg, Sweden

## Abstract

**Background:**

An earlier study from our group of long time survivors of head and neck cancer who had received a low radiation dose to the hypothalamic-pituitary region, with no signs of recurrence or pituitary dysfunction, had their quality of life (QoL) compromised as compared with matched healthy controls. Hippocampal changes have been shown to accompany several psychiatric conditions and the aim of the present study was to test whether the patients’ lowered QoL was coupled to a reduction in hippocampal volume.

**Methods:**

Patients (11 men and 4 women, age 31–65) treated for head and neck cancer 4–10 years earlier and with no sign of recurrence or pituitary dysfunction, and 15 matched controls were included. The estimated radiation doses to the basal brain including the hippocampus (1.5 – 9.3 Gy) had been calculated in the earlier study. The hippocampal volumetry was done on coronal sections from a 1.5 T MRI scanner. Measurements were done by two independent raters, blinded to patients and controls, using a custom method for computer assisted manual segmentation. The volumes were normalized for intracranial volume which was also measured manually. The paired t test and Wilcoxon’s signed rank test were used for the main statistical analysis.

**Results:**

There was no significant difference with respect to left, right or total hippocampal volume between patients and controls. All mean differences were close to zero, and the two-tailed 95% confidence interval for the difference in total, normalized volume does not include a larger than 8% deficit in the patients.

**Conclusion:**

The study gives solid evidence against the hypothesis that the patients’ lowered quality of life was due to a major reduction of hippocampal volume.

## Background

Side effects of high dose radiation therapy directed to the CNS is a well-known concern [1,2]. Less is known about the effects on the brain of low radiation doses, which may result from treatment of cancers outside the CNS, although there is some clinical and laboratory evidence of such effects [3,4]. No studies have as yet with certainty identified human brain regions that are more sensitive to radiotherapy [5,6] but the hippocampus has recently emerged as one possible such region. Cognitive impairment and lowered quality of life are significant sequels in patients irradiated for head and neck tumors and vascular damage resulting in hypoxia in the medial temporal lobe is a possible cause [7,8]. Further, the hippocampus is a neurogenic region of the brain, with the presence of both progenitor cells and a microenvironment suitable for production of new neurons [9]. Children with a slowed cognitive development after adapted radiotherapy treatment of medulloblastoma also had a delayed development of their hippocampi [10,11]. Animal studies have shown that when brains of young rats are unilaterally irradiated, the volume of the irradiated hippocampus is reduced compared to the non-irradiated side, corresponding to an apoptosis-induced loss of proliferating neural stem and progenitor cells [12,13]. A post-mortem study on patients treated with chemotherapy and cranial irradiation, some with reported memory deficits, showed profoundly reduced hippocampal neurogenesis. This further supports the hypothesis that neurocognitive impairment after CNS-directed therapy to some degree is due to a hampered hippocampal neurogenesis [14,15]. A recent laboratory study of 10 Gy radiation to the rodent hippocampus showed significant changes in spine density and morphology in *cornu ammonis 1* beside the changes in the neurogeneous *gyrus dentatus*[16]. There is also experimental evidence that late effects involve yet other areas; one study found that mice with radiation damage to the neurogenic zones had impaired recovery from later ischemic damage [17].

Radiotherapy to patients with cancer in the head and neck region will result in a low dose to the basal parts of the brain. In a recent retrospective study from our group [18] fifteen long-term survivors of such treatment, with no sign of recurrence or pituitary dysfunction, were identified and compared with 15 controls matched for age, sex, BMI and social status. Several quality of life dimensions were significantly compromised in patients compared to controls, an observation which might be related to a negative effect on the CNS of the radiation therapy. Hippocampal volumetry has proven to be a sensitive indicator of several CNS disorders, including Alzheimer’s disease and its precursor states [19,20]. However, to our knowledge no volumetric study of the hippocampi has been performed in patients who have received low-dose radiation to the basal brain at adult age. The purpose of the present study is to test the hypothesis that the lowered quality of life of the patients is due to a substantial reduction in hippocampal volume.

## Methods

### Patients

In 2002, 101 individuals treated for head and neck malignancies were identified from the local database of the Department of Oncology. They had received radiotherapy to the neck and base of the skull during 1992 to 1998 due to cancer in the epipharynx or oropharynx. Out of these 101, fifteen patients (11 men and 4 women, mean age 56 years, range 31–65) with no sign of recurrence participated in a final intensive study. Thirteen of these were treated for cancer of the oropharynx and two for cancer of the epipharynx; the two latter received higher radiation doses to the brain (see below). In order to eliminate several confounders, patients included were highly selected well functioning patients without hypopituitarism due to the radiotherapy and without concomitant somatic disease. For details of the selection process see our companion paper [18]. Median time from radiation treatment to the performance of the study was 6 years (range 4–10 years). None of the selected 15 patients had a significant growth hormone deficiency or other endocrine disturbance but 6 had thyroxin substitution since at least 6 months at the time of the study. Fifteen healthy controls matched for age, sex and BMI were recruited. Relatives or close friends were selected in the first place in order to adjust for social status. The anamnestic investigation of both patients and controls included an estimation of lifetime smoking. One male and one female patient, both in the oropharynx cancer group, were left-handed as were two male controls. Patients and controls all underwent an MRI examination of the brain on a Philips Gyroscan Intera 1.5 T scanner. Written informed consent was received from all participants in the study. The study was approved by the Ethics Committee of the University of Gothenburg (dnr S644-01).

### Radiation treatment and dose to the basal brain

All patients had received external-beam radiotherapy (EBRT) with a beam quality of 4–6 MV from linear accelerators (Varian) using CT-assisted 3-D dose planning (Cadplan System). Thirteen of the patients had also received a brachytherapy boost after the external therapy. For dosage and other details see [18]. In that study, the dose to the pituitary and hypothalamus, including the contribution from the external radiotherapy as well as from the brachytherapy, was calculated in detail from the CT dose plans. The calculations showed that for the 13 patients with cancer of the oropharynx, the median accumulated dose to the hypothalamus was 1.9 Gy (range 1.5-2.2 Gy) and the median dose to the pituitary gland 2.4 Gy (range 1.8-3.3 Gy). The two patients with epipharynx cancer received 9.3/6.0 Gy in the hypothalamus and 46.1 Gy/ 33.5 Gy in the pituitary region. The hippocampi were not clearly demarcated on the CT dose plans and therefore no separate calculation for the dose to the hippocampi was performed. Instead the hippocampi were assumed to receive a similar dose as the hypothalamus since these structures are at a similar distance from the field border.

### Quality of life

In [18], quality of life was assessed using three generic self-rating questionnaires: the Nottingham Health Profile (NHP I) [21], the Psychological General Well-Being (PGWB) index [22], the Symptom Checklist-90 (SCL-90 R) [23] and the Baecke Questionnaire [24]. The patients selected had a lower quality of life, with more anxiety and depressiveness and lower vitality, than the matched controls.

### Hippocampal volumetry

The study was done while a custom method for computer assisted manual volumetry was being developed using the present sample and three other datasets. Only the results from the fully developed method will be reported here. For details about the method see also [19].

The segmentation was performed on interactive Wacom™ PL400 and PL700 screens in the *Hipposegm* routine – a software developed in Matlab™. Before segmentation the MR images were preprocessed using image intensity normalization and Bayesian noise reduction [25]. The noise reduction was performed using bilateral filtering [26,27] with Gaussian kernels.

The hippocampal segmentation was done on T1 weighted coronal slices scanned perpendicularly to the hippocampal principal axis. The main scan parameters for this series and the sagittal series used for ICV segmentation (see below) are presented in Table 1.

**Table 1:** Scan parameters

**Acquisition sequence**		**3D T1 FFE**	**T2 W/TSE**
Orientation		Coronal	Sagittal
Slice thickness	mm	2.4	5
Slice center-to-center distance	mm	1.2	6
Repetition time	ms	25	5834
Echo time	ms	4.6034	110
Flip angle	°	30	90
Field of view	mm	230	250
Acquisition voxel size (AP * LR * FH)	mm^3^	2.4*0.72*0.57	1.12*5.0*0.89
Reconstruction matrix size		512*512	256*256
Reconstruction pixel size	mm^2^	0.45*0.45	0.98*0.98

**Figure 1:**
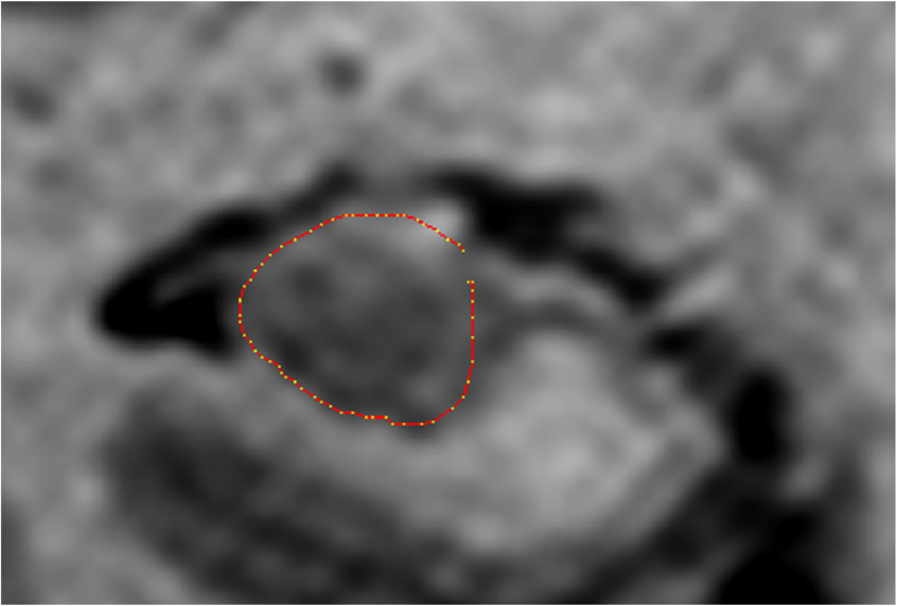
Segmentation of the hippocampal body, including the parts of subiculum contiguous with the hippocampus.

Anatomical definitions of the hippocampus and the hippocampal formation are given in Duvernoy’s sectional anatomy of the hippocampus [28] which is the basis for the segmentation protocol used. Our protocol is partly similar to that of Convit [29,30] and only the part of the subiculum inferior of and contiguous with the hippocampus was included (Figure 1). The fimbria and fornix were excluded, and the hippocampal tail segmentation was based on Maller [31]. Since limited resolution makes it difficult to demarcate the alveus from other parts of the hippocampus on 1.5 T scanners [32], it was included in the segmentation.

Table 2 summarizes the rules adhered to in the present study.

**Table 2:** Definitions of hippocampal segmentation borders

	
Anterior border	Landmark setting where the uncal recess of the temporal horn or the alveus is visible in the sagittally reformatted image
Posterior border	Landmark setting between the gray matter of the hippocampal tail and the surrounding white matter in the sagittally reformatted image
Medial border	Border between the hippocampal body and the transverse fissure; border between the hippocampal head and the crural cistern
Lateral border	Medial wall of the temporal horn
Inferior border	Border between the gray matter of the subiculum and the white matter in the parahippocampal gyrus

**Figure 2:**
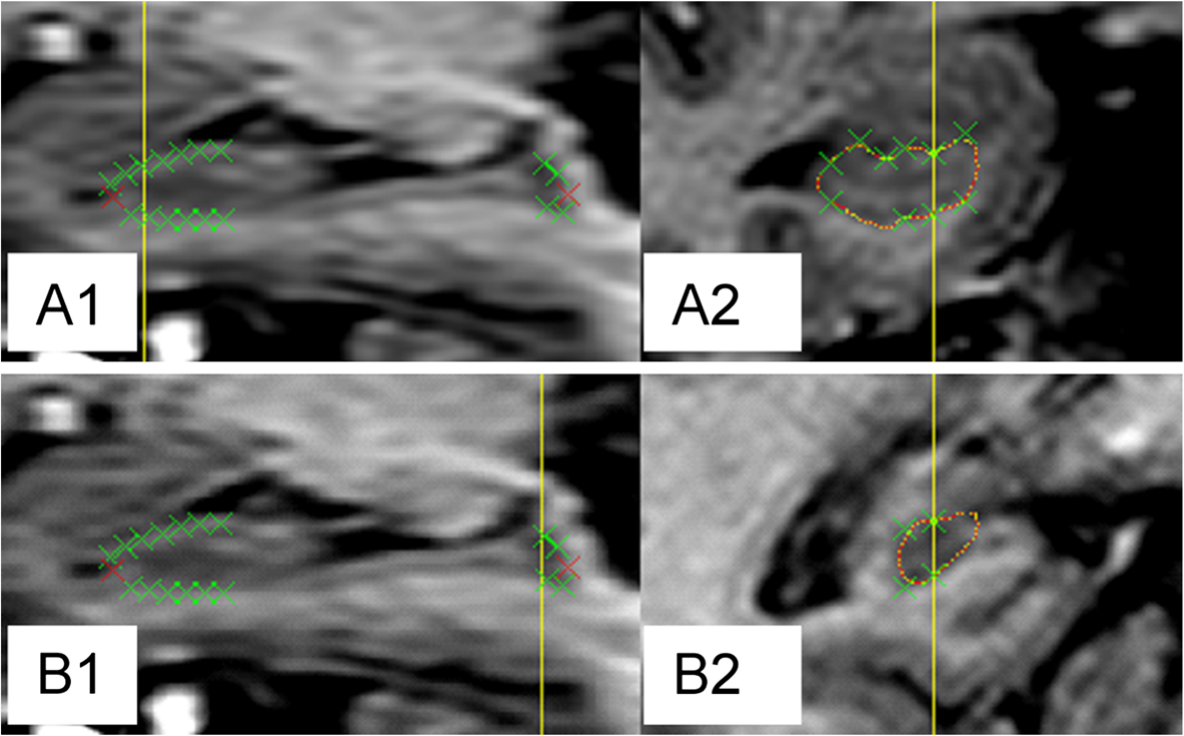
**Landmark setting. A1.** Landmarks set in the reformatted sagittal slice of the hippocampal region. Yellow line shows the position of the coronal slice to in A2. Red crosses used for anterior and posterior limits. Green crosses used for other limits. **A2.** Landmarks transformed into the coronal view to guide the segmentation (red line) in the anterior hippocampal head. **B1.** Landmarks set in the reformatted sagittal slice of the hippocampal region. Yellow line showing the position of the coronal slice in B2. **B2.** Landmarks (crosses) transformed into the coronal view to guide the segmentation (red line) in the most difficult parts of the hippocampal tail.

The segmentation process consisted of two steps: 1. Pointwise landmark setting was done in the reformatted sagittal view of the coronal images where the demarcation in the original coronal images is indiscernible or difficult to interpret. 2. Segmentation of the hippocampus in the coronal images was done by continuous pen drawing. By means of the landmark setting and noise reduction, the whole hippocampus including the tail [31] could be segmented without ad hoc determination of the most anterior and the most posterior slice [33,34].^a^ See Figure 2.

A 3D-visualisation was done after the preliminary segmentation of the hippocampus to check for deviations from anatomical and curvature expectations.

Two raters, EO and CE, both segmented the whole material using the fully developed method. Both raters were blind for group belonging, patient ID, and other patient data. Because of EO’s greater experience with the method, we have chosen to present the results from his measurement. (CE’s measurements gave quite similar results).

### Intracranial volume estimation and normalization

To reduce the variance in hippocampal volumes by normalization to skull size, intracranial volumes (ICV) were measured for all subjects. Since we were at the time developing and validating a quick algorithm for estimating ICV, the same two raters (EO and CE) did a full manual segmentation of ICV in the whole material, using the *Hipposegm* software on 5 mm T2 sagittal slices. The mean of these measurements was used as the value of ICV. The main scan parameters for the ICV segmentation are summarized in Table 1 above. No results from the ICV measurement are presented here except its inter-rater reliability.

We then calculated the regression of left and right hippocampal volumes on ICV in the whole material. To get a normalized volume V_norm_ from an absolute volume V_abs_, we used the formula [35]:

$${\text{V}}_{\text{norm}}={\text{V}}_{\text{abs}}–\text{k}*\left(\text{ICV}–\text{Mean}\left(\text{ICV}\right)\right)$$

where ICV is the current ICV estimate, k is the detected regression coefficient and Mean(ICV) refers to the mean estimated ICV in the material. Mean normalized volume in the whole sample therefore equals mean absolute volume.

### Statistics

Interrater reliability for the hippocampal segmentation by the two raters was calculated using raw correlation (Pearson’s r) and intraclass correlation (ICC, two-way mixed model, single measure reliability, both absolute agreement and consistency versions).

Interrater reliability for the intracranial volume segmentation by the two raters was calculated using Pearson’s r and ICC (two-way mixed model, average measure reliability, absolute agreement).

Pairwise comparisons of normalized hippocampal volumes (left and right side separately, as well as total volumes) between patients and their matched controls were performed using both parametric and non-parametric methods: paired *t* test (two-tailed) with 95% confidence intervals and Wilcoxon’s signed rank test. Hippocampal volumes on the right and the left side were compared for all subjects using the same tests. Groupwise comparisons between male and female subjects with respect to both absolute and normalized hippocampal volumes were also performed. For these, the unpaired *t* test (two-tailed) was used together with Mann-Whitney’s *U* test. Before the unpaired *t* test the homogeneity of variances was tested with Levene’s test of equality of variances. Beside the paired and unpaired comparisons, hippocampal and intracranial volumes were correlated with age. For correlations, Pearson’s r and Spearman’s ρ were used.

Since the results using parametric and non-parametric methods were generally in very good agreement, only those from the parametric methods are reported.

Calculations were done on the whole sample of 30 subjects and, in order to maximize the homogeneity of the sample, also on a restricted group that did not include the two patients with epipharyngeal cancer (and in the pairwise comparisons, their controls).

The main calculations were made using StatView 5.0 for Macintosh. For the reliability analysis, SPSS 19 for Macintosh was also used.

## Results

### Reliability

The raw correlation (Pearson’s r) between the two raters’ measurements of intracranial volume was 0.987 and the absolute agreement intraclass correlation (ICC; two-way mixed model, average measure reliability) was 0.992.

The raw correlation (Pearson’s r) between EO’s and CE’s measurements of total hippocampal volumes was 0.854; the absolute agreement intraclass correlation (ICC; two-way mixed model, single measure reliability) was 0.764. Consistency ICC (two-way mixed model, single measure reliability) was 0.852.

### Main results

Table 3 shows the results of a pairwise comparison of normalized hippocampal volumes between patients and controls in the restricted homogeneous sample of 13 pairs. A negative difference means that the patient mean is below the control mean.

**Table 3:** Normalized right, left and total hippocampal volumes (mm^3^) in 13 patients and their controls^2^

	**Pat mean**	**Cont mean**	**Diff**	**DF**	***t *value**	***p***	**95% CI**
Right	2454.8	2476.1	−21.3	12	−0.186	0.839	−244.4 < D < 201.9
Left	2324.5	2265.8	58.7	12	0.774	0.525	−136.4 < D < 253.9
Total	4779.3	4741.8	37.4	12	0.272	0.840	−357.5 < D < 432.5

^2^Pat mean: Patient mean normalized volume. Cont mean: Control mean normalized volume. Diff: difference between patient and control mean. DF: degrees of freedom. *p*: significance level of patient/control difference, paired t test (two-tailed). CI: Confidence interval for difference between patient and control mean.

The comparison does not reveal any significant difference in any of the measures of normalized hippocampal volumes. The observed small mean patient/control volume differences – at most 3% of a mean volume – go both ways. The two-tailed 95% confidence interval for the difference in total normalized hippocampal volume, expressed as a percentage of the volume mean, ranges from 7.5% on the negative side (corresponding to smaller patient volumes) to 9.0% on the positive side (corresponding to larger patient volumes). Adding the two epipharynx patients (see Table 4) to the sample does not change the results notably except that it further compresses the confidence interval. A statistical subgroup analysis based on gender is not meaningful because of the low number of female participants.

**Table 4:** Mean absolute and normalized hippocampal volumes (mm^3^) in the restricted group of 28 subjects, split on men and women, and of the two epipharynx patients^3^

	**Right abs**	**Left abs**	**Total abs**	**Rightnorm**	**Leftnorm**	**Total norm**	**R/L *p***
All (n = 28)	2472.2 ± 676.9	2301.1 ± 622.7	4773.3 ± 1261.5	2466.7 ± 519.8	2296.1 ± 456.0	4762.7 ± 924.6	<0.0001
Men (n = 21)	2530.9 ± 689.3	2364.9 ± 659.6	4895.8 ± 1310.4	2458.7 ± 519.6	2299.5 ± 500.5	4758.2 ± 968.3	
Women (n = 7)	2296.0 ± 534.2	2109.8 ± 258.6	4405.9 ± 773.4	2490.4 ± 559.0	2285.9 ± 316.7	4776.3 ± 848.6	
M/W *p*			0.028*			0.931	
Pat 4 W	2167.3	2268.7	4436.0	2430.4	2507.1	4937.5	
Pat 6 M	2463.1	2009.6	4472.6	2355.1	1911.8	4266.8	

^3^Group results are presented plus minus 2 standard deviations. Right abs: absolute volume of right hippocampus, etc. Rightnorm: normalized volume of right hippocampus, etc. R/L *p*: significance level of the difference between right and left volumes (paired t test). M/W *p*: significance level of the differences between.

### Comparisons of men and women, and left vs right hippocampus

The mean absolute and normalized hippocampal volumes in the restricted group of 28 subjects, split on men and women, are presented in Table 4. Since the results of these calculations were similar in patients and controls, they are not presented separately. The last two rows of Table 4 present the corresponding results from Patient 4 (man) and Patient 6 (woman), both with former epipharyngeal cancer. Note that the measure of variation is 2 standard deviations.

There is a nearly significant difference, in the order of 10%, between men and women regarding total absolute volumes. The difference is eradicated when the volumes are normalized. There is also a highly significant absolute volume difference of 7.1% between left and right hippocampus in the group of 28. Patients vs control data are not shown in Table 4 but the L/R difference was somewhat higher in the control group (8.5%) and smaller (5.5%) but still highly significant among the patients. Also not shown is that among the 24 right-handed subjects in the restricted sample the L/R difference was 6.7%, and among the four left-handed subjects it was 9.5% (the right hippocampus still the bigger one).

It should be noted that patient 4 had a very small ICV compared to patient 6. In terms of normalized volumes, the left hippocampus of both patients deviate somewhat from the mean of the restricted sample (cf. Table 3). Patient 4 lies one SD *above* the group mean, while patient 6 lies one and a half SD below the mean and has the next to lowest normalized left hippocampal volume in the whole sample of 30. Their right hippocampal volumes are close to the restricted group mean.

### The hippocampus and age

The correlation between age and total normalised hippocampal volume in the whole sample is negative (−0.457) and significant (p = 0.0103). In the restricted sample of 28 it is still significant (p = 0.0221). If the restricted sample is split according to gender, the correlation becomes −0.500 (p = 0.0198) in the male group, but is close to zero (actually weakly positive) among the females. The correlation is essentially the same among the male patients (−0.499) and the male controls (−0.513). The age change in the male group corresponds to an annual 0.5% decrease in volume.

## Discussion

This is to our knowledge the first study on hippocampal volumes after low dose radiation to the basal part of the adult human brain. Although the dose to the hippocampus could not be calculated directly, the estimates of radiation dose to relevant areas are probably more exact than in any previous study. The small study size is an effect of our ambitions to minimize the influence of confounding factors in an original sample of 101 patients. Moreover, the patient sample is homogeneous in terms of treatment with the exception of two patients who received a higher radiation dose; these were treated separately in the statistical analysis.

Automatic methods for hippocampal volumetry are rapidly gaining acceptance. They have undisputed advantages in terms of cost, inter-rater reliability and comparability between studies. However, for small-scale studies involving only intra-study comparisons, we would argue that manual segmentation is still superior. This is even more so since the issue of ICV normalization has not been satisfactorily resolved for the most used automatic method [36].

The reliability results for the volumetric method are acceptable. Since the main results in the study are based on differences between patients or groups, the most relevant measure when comparing the results of the two raters is consistency ICC which does not take systematic (non-random) differences between the raters into account. Importantly, consistency ICC was considerably higher than absolute measure ICC which reflects that the latter was strongly influenced by such a systematic difference. When interpreting the reliability figures, one should also bear in mind that the method included segmentation of the hippocampal tail, which is the most difficult part and adds variation in comparison with not including the tail (data not shown).

The size of the observed interindividual variation in hippocampal volumes as reported in Table 4 accords with recently published data from healthy subjects [37]. Other facts that speak in favour of the validity of our measurements are that the observed volumetric differences between left and right hippocampal volumes and between men and women, as well as the negative correlation with age in the male group, are in general accord with main trends among earlier findings [31,36,38,39]. The L/R difference and the age correlation were similar in patients and controls. The influence of handedness could not be tested properly since the number of left-handed subject was too low to admit any statistically meaningful subgroup calculation.

Intracranial volume, ICV, was measured with a highly reliable manual method. Normalization of hippocampal volumes with respect to ICV eliminated the gender differences and reduced the overall variance. Somewhat surprisingly, the latter does not hold for the female group. This is probably a statistical artifact since there were only seven females while the normalization was based on a regression in the whole sample of 30 subjects.

It could be argued that the sample is small and that the study therefore has insufficient power. This argument would have had a point if our only result had been that the mean volume difference between patients and controls was not significantly different from zero. However, all observed mean volume differences were close to zero, and the 95% confidence interval for the difference in total normalized volume does not include larger deficits in the patient group than 8% of the group mean. Using the data from the second rater would have given very similar results. Hence our results constitute solid positive evidence that low dose radiation to the basal brain in adults does not cause a lasting, major volume reduction of the hippocampi. The lowered quality of life in our patient group stands in need of some other explanation.

The neurogenic cells in the *gyrus dentatus* are the most radiosensitive elements of the hippocampus and a subregion analysis would have added important information. However, such an analysis is not feasible on 1.5 T data. Further, experimental and clinical evidence support the thought that the cognitive effects seen long after low dose radiation to the brain are at least partly mediated by indirect effects on other structures than the *gyrus dentatus*[40].

The method described in this work may be of value in the future considering the change in radiation treatment techniques that are being introduced, such as IMRT (intensity modulated radiation therapy) and SRT (stereotactic radiation therapy). The radiation dose will be better targeted and controlled with these techniques, but the areas receiving low dose will be much larger than after the traditional methods used in our study. It is not known what this means biologically and clinically, and it has to be studied and documented carefully. Our study should be seen as a part of this work.

## Endnote

^a^In two subjects the scans were incomplete at the level of the tail and mean tail values had to be imputed.

## Competing interests

The authors have no conflict of interest that could be perceived as prejudicing the impartiality of the research reported.

## Authors' contributions

EO, CE, EL, GJ, SE and HM contributed to the conception and the design of the trial and drafted the first version of the manuscript. All authors contributed to the collection of data, data interpretation and critical revision of the manuscript and have reviewed the final version for publication.
